# Using a 31-Gene Expression Profile Test to Stratify Patients with Stage I–II Cutaneous Melanoma According to Recurrence Risk: Update to a Prospective, Multicenter Study

**DOI:** 10.3390/cancers14041060

**Published:** 2022-02-19

**Authors:** Sebastian Podlipnik, Aram Boada, Jose L. López-Estebaranz, Manuel M. Martín-González, Pedro Redondo, Brian Martin, Ann P. Quick, Christine N. Bailey, Sarah J. Kurley, Robert W. Cook, Susana Puig

**Affiliations:** 1 Department of Dermatology, Hospital Clinic of Barcelona, 08036 Barcelona, Spain; podlipnik@clinic.cat (S.P.); spuig@clinic.cat (S.P.); 2 Department of Dermatology, Hospital Universitari Germans Trias i Pujol, Institut d’investigació Germans Trias Badalona, 08916 Barcelona, Spain; aboada.germanstrias@gencat.cat; 3 Department of Dermatology, Hospital Universitario Fundación Alcorcón, Alcorcón, 28922 Madrid, Spain; jlestebaranz@salud.madrid.org; 4 Department of Dermatology, Hospital Universitario Ramón y Cajal, 28034 Madrid, Spain; manuelmaria.martin@salud.madrid.org; 5 Department of Dermatology, University Clinic of Navarra, 31008 Pamplona, Spain; predondo@unav.es; 6 Castle Biosciences, Inc., Friendswood, TX 77546, USA; bmartin@castlebiosciences.com (B.M.); aquick@castlebiosciences.com (A.P.Q.); cbailey@castlebiosciences.com (C.N.B.); skurley@castlebiosciences.com (S.J.K.); 7 Department of Dermatology, University of Barcelona, IDIBAPS, 08007 Barcelona, Spain; 8 Centro de Investigación Biomédica en Red de Enfermedades Raras, CIBERER, Instituto de Salud Carlos III, 46010 Barcelona, Spain

**Keywords:** 31-gene expression profile, stage I–II, cutaneous melanoma, personalized medicine, prospective

## Abstract

**Simple Summary:**

Many people with skin cancer will have their cancer come back. The 31-gene expression profile (31-GEP) test can help predict if a cancer has a low (Class 1) or high (Class 2) chance of returning. This study looked at 86 patients with early skin cancer to see how well the 31-GEP test predicted if their cancer would return. None of the patients with a Class 1 GEP result had their cancer return within 3 years, but one-fourth of patients with a Class 2 result did. This study showed that the 31-GEP test can help predict if a patient’s skin cancer will return. Accurate risk prediction can help doctors make better treatment plans for patients with skin cancer.

**Abstract:**

Background: Fifteen to forty percent of patients with localized cutaneous melanoma (CM) (stages I–II) will experience disease relapse. The 31-gene expression profile (31-GEP) uses gene expression data from the primary tumor in conjunction with clinicopathologic features to refine patient prognosis. The study’s objective was to evaluate 31-GEP risk stratification for disease-free survival (DFS) in a previously published cohort with longer follow-up. Methods: Patients with stage IB–II CM (*n* = 86) were prospectively tested with the 31-GEP. Follow-up time increased from 2.2 to 3.9 years. Patient outcomes were compared using Kaplan-Meier and Cox regression analysis. Results: A Class 2B result was a significant predictor of 3-year DFS (hazard ratio (HR) 8.4, *p* = 0.008) in univariate analysis. The 31-GEP significantly stratified patients by risk of relapse (*p* = 0.005). A Class 2B result was associated with a lower 3-year DFS (75.0%) than a Class 1A result (100%). The 31-GEP had a high sensitivity (77.8%) and negative predictive value (95.0%). Conclusions: The 31-GEP is a significant predictor of disease relapse in patients with stage IB–II melanoma and accurately stratified patients by risk of relapse.

## 1. Introduction

Cutaneous melanoma (CM) incidence has been increasing steadily since 1980, while CM mortality has decreased [1,2,3].One reason for this may be melanoma diagnoses at earlier tumor stages but also due to the introduction of new systemic treatments during the last decade [4]. Patients with stage I–II CM have localized disease, while stage III CM is characterized by the presence of microsatellites or regional metastasis, and stage IV by distant metastasis. Most newly diagnosed CM patients are diagnosed with localized, early stage I–II CM [4,5]. Despite overall good outcomes for many patients with localized disease (98% and 90% 5-year melanoma-specific survival for stage I and II, respectively) [6], 15% of stage I and 40% of stage II patients will still experience a recurrence or metastasis and may ultimately die from their disease [7]. Therefore, additional methods for early identification of which tumors are most likely to metastasize could aid in better patient management for better long-term outcomes.

We previously reported results from a prospective study using a gene expression profile test (31-GEP) that uses the expression of discriminating and control genes from the primary tumor to stratify patients with stage IB–II CM by recurrence risk [8]. The 31-GEP classifies patients as being at low (Class 1) or high (Class 2) risk of recurrence or distant metastasis and can further classify them as having the lowest (Class 1A), intermediate (Class 1B/2A), or highest (Class 2B) risk [9,10,11,12,13]. Despite a relatively short follow-up of 2.2 years in the previously published study, patients with a Class 2 result experienced recurrences and had lower survival rates than patients with a Class 1 result, who did not experience recurrences. The 31-GEP was an independent predictor of disease-free survival (DFS) [8]. Therefore, the objective of this study was to report updated DFS stratification according to 31-GEP classes (1A, 1B/2A, 2B) for longer-term outcomes to confirm the validity of the 31-GEP test in the study population.

## 2. Materials and Methods

### 2.1. Study Design

The study design has been reported previously [8]. Briefly, patient data were collected prospectively from five melanoma referral centers in Spain (April 2015–December 2016) and staged according to AJCC 7th edition criteria (*n* = 86). American Joint Committee on Cancer (AJCC) establishes the criteria used to stage patients with CM based on tumor thickness and ulceration (and mitotic rate for AJCC 7th edition used at the time of this study), as well as regional and distant metastasis [6]. All patients underwent a sentinel lymph node biopsy surgical procedure, and only those patients pathologically staged as stage IB–II were included.

Because we obtained longer follow-up (median: 3.9 years vs. 2.2 years) for these patients, the primary outcome was three-year DFS, which assessed the time from diagnosis to relapse—including local, regional, and distant disease recurrences—in the study population. Each participating hospital’s ethics committee approved the study.

### 2.2. 31-GEP Testing

The 31-GEP has been discussed in detail previously [9]. Briefly, the 31-GEP analyzes gene expression of 28 discriminating gene targets across 27 genes and three control genes from formalin-fixed, paraffin-embedded primary tumor tissue. While we previously reported outcomes based on main class (Class 1 vs. Class 2), we report three risk groups in this update to show low (Class 1A), intermediate (Class 1B/2A), or high (Class 2B) risk groups for more precise risk stratification. Further, supplemental data shows risk stratification for the 31-GEP subclass and main class to confirm previous results.

### 2.3. Statistical Analysis

Patient demographics were compared using the Kruskal-Wallis test for continuous variables and Pearson’s Chi-square test for categorical variables. Survival analysis was performed using Kaplan-Meier analysis with the log-rank test. Accuracy metrics were calculated in two ways: first, by using Class 1A as a negative result and Class 2B as a positive result, and second by considering Class 1 and Class 2 as a negative and positive result, respectively. Univariate Cox regression was used to determine the ability of the 31-GEP and AJCC staging (7th edition and we restaged to the 8th edition) to predicted disease relapse.

## 3. Results

Patient demographics are shown in Table 1. There was no significant difference between groups for age (*p* = 0.133) or sex (*p* = 0.253). Class 1A patients who experienced disease relapse (*n* = 2) had a longer time to recurrence (median 3.8 years) than those with a Class 1B/2A (*n* = 3; median 1.8 years) or a Class 2B result (*n* = 7; median 1.1 years) though this did not reach significance (*p* = 0.283). There was a significant difference between groups for Breslow thickness (Class 1A [1.3 mm] vs. Class 1B/2A [2.0 mm] vs. Class 2B [3.5 mm], *p* < 0.001), number of mitoses (Class 1A [1/mm^2^] vs. Class 1B/2A [3/mm^2^] vs. Class 2B [8/mm^2^], *p* < 0.001), and presence of ulceration (Class 1A [2.5%, 1/40] vs. Class 1B/2A [40.0%, 10/25] vs. Class 2B [71.4%, 15/21], *p* < 0.001).

**Figure 1:**
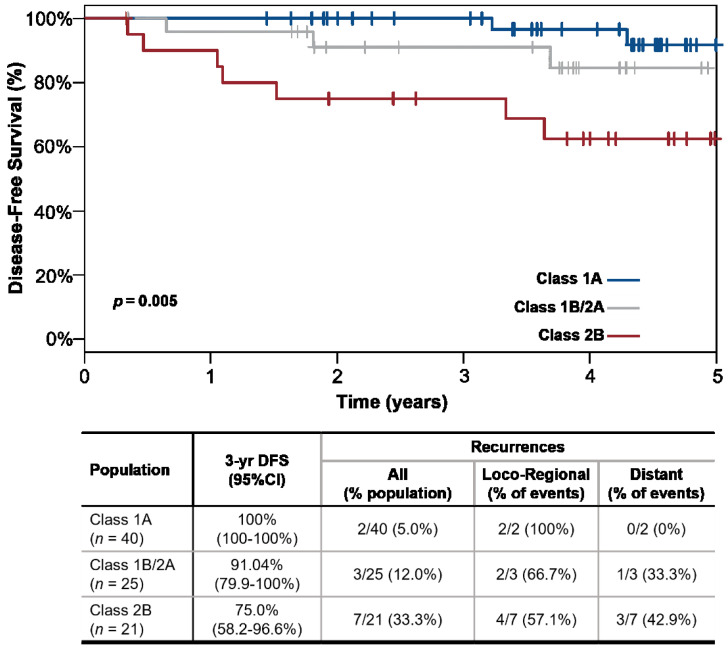
The 31-GEP stratifies patients by 3-year disease-free survival (DFS). Kaplan-Meier (KM) Curve. Patients with a Class 1A 31-GEP result (blue line) have higher 3-year DFS than patients with a Class 1B/2A (orange line) or Class 2B (red line) result. Patients with a 31-GEP Class 1A result had lower numbers of recurrences and no distant metastases than patients with a Class 1B/2A or Class 2B result. CI: confidence interval.

Of the recurrences experienced by patients with a Class 1A result, one of two recurrences was local, one was an in-transit metastasis, and no Class 1A recurrences were distant metastases. In contrast, of the disease recurrences experienced by patients with a Class 2B result, none were local recurrences, 4/7 (57.1%) were regional, and 42.9% (3/7) were distant metastases (Figure 1). Kaplan-Meier analysis showed that the 31-GEP significantly stratified patients by their risk of disease relapse (*p* = 0.005). Patients with a Class 1A result had higher 3-year DFS (100%) than patients with a Class 1B/2A (91.0%) or a Class 2B (75.0%) result (Figure 1). Further, the 31-GEP significantly stratified risk when analyzed as 31-GEP subclass (Class 1A, 1B, 2A, 2B, *p* = 0.01) and 31-GEP main class (Class 1, Class 2, *p* = 0.004) (Figures S1 and S2). Univariate cox regression analysis showed that a 31-GEP Class 2B result was a significant predictor of 3-year DFS (HR 8.4 [95% CI 1.7–40.7], *p* = 0.008) (Table 2). Finally, the 31-GEP had a high sensitivity (77.8%) and negative predictive value (NPV: 95.0%), and patients with a Class 2B result had nearly three times the odds of relapse than patients with a Class 1A result (positive likelihood ratio: 2.9; Table 3).

## 4. Discussion

Early-stage localized (stage I–II) melanoma accounts for most newly diagnosed CM cases, and due to a large number of cases relative to higher stages, most CM-related deaths each year occur in the patient population initially diagnosed with low-stage melanoma [4,5]. Reports suggest that individuals who die from melanoma lose up to 20.4 years of potential life on average and that the annual cost of melanoma treatment in the United States will reach $1.6 billion by 2030 [14]. Therefore, the identification of patients who are likely to experience disease relapse (or not) is necessary to aid in earlier detection of recurrence or metastasis, reduce unnecessary treatment and focus healthcare resources to patients most likely to benefit.

In this study, the 31-GEP identified patients with stage IB–II CM who had a higher risk of disease relapse (Class 2B) than patients classified as lowest risk (Class 1A) by the 31-GEP. The high NPV of the test suggests that patients who receive a low-risk 31-GEP result are unlikely to experience adverse effects of reduced treatment intensity. In contrast, a high sensitivity suggests that the test can identify a majority of patients who will experience disease relapse and should be monitored more closely.

In addition to Class 1A having a low relapse rate (*n* = 2), none were distant, consistent with prior studies of the two patients with a Class 1A result who did experience a relapse [10,15]. In contrast, patients with Class 1B/2A or Class 2B results had median recurrence times of less than two years (1.8 and 1.1 compared with 3.8 for Class 1A), and follow-up intensity early on for these patients may help identify recurrences or metastases earlier. These data suggest that using the 31-GEP can help identify those patients most likely to benefit from increased disease surveillance intensity or methods.

A limitation of the study is that relatively few events limited Cox regression to univariate analysis for DFS. However, previous studies on the 31-GEP have consistently shown that the 31-GEP is a significant independent predictor for relapse [10,11,12,13]. The primary purpose of the current analysis was to provide longer follow-up time to confirm the results from the previous publication. The results presented here indicate low-risk patients continue to do well, beyond 2 years of follow up. To maintain consistency with the previous report, we report results based on AJCC 7th edition staging; however, restaging to AJCC 8th edition did not change risk stratification results by the 31-GEP, and univariate analysis was only mildly changed for AJCC staging. Further, the low number of patients may limit the interpretation and generalizability of the results. Nevertheless, the data support the conclusions from the earlier analysis [8], are consistent with several other independent studies [11,16], and show that the 31-GEP can accurately identify patients at low or high risk of disease relapse.

## 5. Conclusions

The 31-GEP accurately identified patients with stage IB-II CM at low and high risk of melanoma relapse in a prospective, multicenter study. Thus, identifying which tumors have the highest probability of recurrence or metastasis, despite patients having a negative SLN can aid in earlier disease detection.

## Figures and Tables

**Table 1:** Patient Demographics.

Clinicopathologic Feature	Class 1A (*n* = 40)	Class 1B/2A (*n* = 25)	Class 2B (*n* = 21)	Combined (*n* = 86)	*p*-Value ^†^
Age (years)					
Median (Range)	58 (26–79)	66 (23–82)	65 (32–86)	60 (23–86)	*p* = 0.133
Sex					
Female	24/40 (60%)	14/25 (56%)	8/21 (38.1%)	46/86 (53.49%)	*p* = 0.253
Male	16/40 (40%)	11/25 (44%)	13/21 (61.9%)	40/86 (46.51%)	
AJCC 7th Ed. Stage					
IB	32/40 (80%)	9/25 (36%)	3/21 (14.29%)	44/86 (51.16%)	*p* < 0.001
IIA	5/40 (12.5%)	10/25 (40%)	3/21 (14.29%)	18/86 (20.93%)	
IIB	3/40 (7.5%)	3/25 (12%)	9/21 (42.86%)	15/86 (17.44%)	
IIC	0/40 (0%)	3/25 (12%)	6/21 (28.57%)	9/86 (10.47%)	
AJCC 8th Ed. Stage					*p* < 0.001
IA	17/40 (43%)	2/25 (8%)	0/21 (0%)	19/86 (22%)	
IB	16/40 (40%)	7/25 (28%)	3/21 (14%)	26/86 (30%)	
IIA	4/40 (10%)	10/25 (40%)	2/21 (10%)	16/86 (19%)	
IIB	3/40 (8%)	4/25 (16%)	11/21 (52%)	18/86 (21%)	
IIC	0/40 (0%)	2/25 (8%)	5/21 (24%)	7/86 (8%)	
Breslow Thickness (mm)					
Median (Range)	1.3 (0.4–7.0)	2.0 (0.8–10)	3.5 (1.1–15.0)	1.7 (0.4–15.0)	*p* < 0.001
Mitoses (1/mm^2^)					
Median (Range)	1 (0–6)	3 (0–12)	8 (0–32)	2 (0–32)	*p* < 0.001
Ulceration					
Absent	39/40 (97.5%)	15/25 (60%)	6/21 (28.57%)	60/86 (69.77%)	*p* < 0.001
Present	1/40 (2.5%)	10/25 (40%)	15/21 (71.43%)	26/86 (30.23%)	
Disease Relapse					
No	38/40 (95%)	22/25 (88%)	14/21 (66.67%)	74/86 (86.05%)	*p* = 0.009
Yes	2/40 (5%)	3/25 (12%)	7/21 (33.33%)	12/86 (13.95%)	
Follow-up (years)					
Median follow up with no relapse, years, median (range)	4.2 (1.4–5.3)	3.8 (0.3–4.9)	4.1 (0.3–5.0)	3.9 (0.3–5.3)	*p* = 0.237
Time to relapse, years, median (range)	3.8 (3.2–4.3)	1.8 (0.6–3.7)	1.1 (0.3–3.6)	1.7 (0.3–4.3)	*p* = 0.283

^†^ Kruskal-Wallis test was used to compare continuous data. Chi-square was used to compare categorical data.

**Table 2:** Cox univariate regression analysis for 3-year DFS.

Feature	Univariate HR (95% CI)	*p*-Value
31-GEP Class 1A 31-GEP Class 1B/2A	reference 2.8 (0.5–17.2)	- 0.253
31-GEP Class 2B	8.4 (1.7–40.7)	0.008
Stage I–IIA	reference	-
Stage IIB–IIC (V7)	8.8 (2.4–32.6)	0.001
Stage IIB–IIC (V8)	8.5 (2.3–31.4)	0.001

HR: hazard ratio; V7: American Joint Committee on Cancer melanoma staging, version 7; V8: American Joint Committee on Cancer melanoma staging, version 8.

**Table 3:** Accuracy metrics for the 31-GEP. Stage IB–II (*n* = 86).

Metric Class 1A vs. Class 2B	%, (95% CI)	Likelihood Ratios
Sensitivity	77.8% (40.2–96.1%)	Positive: 2.9 (1.6–5.1)
Specificity	73.1% (58.7–84.0%)	
PPV	33.3% (15.8–56.9%)	Negative: 0.3 (0.1–1.0)
NPV	95.0% (81.8–99.1%)	
**Metric** **Class 1 vs. Class 2**	**%, (95% CI)**	**Likelihood Ratios**
Sensitivity	75.0% (42.8–93.3%)	Positive: 2.3 (1.5–3.7)
Specificity	67.6% (55.6–77.7%)	
PPV	27.3% (13.9–45.8%)	Negative: 0.4 (0.1–1.0)
NPV	94.3% (83.4–98.5%)	

PPV: positive predictive value; NPV: negative predictive value; CI: confidence interval.

## Data Availability

Patient data will not be made publicly available.

## Supplementary Materials

The following are available online at https://www.mdpi.com/article/10.3390/cancers14041060/s1, Figure S1: The 31-GEP stratifies patients by 3-year disease-free survival (DFS) when analyzed by 31-GEP subclass, Figure S2: The 31-GEP stratifies patients by 3-year disease-free survival (DFS) when analyzed by 31-GEP main class.

## References

[1] Glazer A.M., Winkelmann R.R., Farberg A.S., Rigel D.S. (2017). Analysis of Trends in US Melanoma Incidence and Mortality. JAMA Dermatol..

[2] American Cancer Society (2020). Cancer Facts & Figures 2020.

[3] Curti B.D., Faries M.B. (2021). Recent Advances in the Treatment of Melanoma. N. Engl. J. Med..

[4] Herbert A., Koo M.M., Barclay M.E., Greenberg D.C., Abel G.A., Levell N.J., Lyratzopoulos G. (2020). Stage-Specific Incidence Trends of Melanoma in an English Region, 1996–2015: Longitudinal Analyses of Population-Based Data. Melanoma Res..

[5] Kwatra S.G., Hines H., Semenov Y.R., Trotter S.C., Holland E., Leachman S. (2020). A Dermatologist’s Guide to Implementation of Gene Expression Profiling in the Management of Melanoma. J. Clin. Aesthet. Dermatol..

[6] Gershenwald J.E., Scolyer R.A., Hess K.R., Sondak V.K., Long G.V., Ross M.I., Lazar A.J., Faries M.B., Kirkwood J.M., McArthur G.A. (2017). Melanoma Staging: Evidence-Based Changes in the American Joint Committee on Cancer Eighth Edition Cancer Staging Manual. CA Cancer J. Clin..

[7] Rockberg J., Amelio J.M., Taylor A., Jörgensen L., Ragnhammar P., Hansson J. (2016). Epidemiology of Cutaneous Melanoma in Sweden—Stage-Specific Survival and Rate of Recurrence. Int. J. Cancer.

[8] Podlipnik S., Carrera C., Boada A., Richarz N.A., López-Estebaranz J.L., Pinedo-Moraleda F., Elosua-González M., Martín-González M.M., Carrillo-Gijón R., Redondo P. (2019). Early Outcome of a 31-Gene Expression Profile Test in 86 AJCC Stage IB-II Melanoma Patients. A Prospective Multicentre Cohort Study. J. Eur. Acad. Dermatol. Venereol..

[9] Gerami P., Cook R.W., Wilkinson J., Russell M.C., Dhillon N., Amaria R.N., Gonzalez R., Lyle S., Johnson C.E., Oelschlager K.M. (2015). Development of a Prognostic Genetic Signature to Predict the Metastatic Risk Associated with Cutaneous Melanoma. Clin. Cancer Res..

[10] Zager J.S., Gastman B.R., Leachman S., Gonzalez R.C., Fleming M.D., Ferris L.K., Ho J., Miller A.R., Cook R.W., Covington K.R. (2018). Performance of a Prognostic 31-Gene Expression Profile in an Independent Cohort of 523 Cutaneous Melanoma Patients. BMC Cancer.

[11] Keller J., Schwartz T.L., Lizalek J.M., Chang E., Patel A.D., Hurley M.Y., Hsueh E.C. (2019). Prospective Validation of the Prognostic 31-gene Expression Profiling Test in Primary Cutaneous Melanoma. Cancer Med..

[12] Hsueh E.C., DeBloom J.R., Lee J.H., Sussman J.J., Covington K.R., Caruso H.G., Quick A.P., Cook R.W., Slingluff C.L., McMasters K.M. (2021). Long-Term Outcomes in a Multicenter, Prospective Cohort Evaluating the Prognostic 31-Gene Expression Profile for Cutaneous Melanoma. JCO Precis. Oncol..

[13] Arnot S.P., Han G., Fortino J., Han D., Fowler G., Vetto J.T. (2021). Utility of a 31-Gene Expression Profile for Predicting Outcomes in Patients with Primary Cutaneous Melanoma Referred for Sentinel Node Biopsy. Am. J. Surg..

[14] Guy G.P., Thomas C.C., Thompson T., Watson M., Massetti G.M., Richardson L.C. (2015). Centers for Disease Control and Prevention (CDC) Vital Signs: Melanoma Incidence and Mortality Trends and Projections—United States, 1982–2030. MMWR Morb. Mortal. Wkly. Rep..

[15] Gastman B.R., Gerami P., Kurley S.J., Cook R.W., Leachman S., Vetto J.T. (2019). Identification of Patients at Risk of Metastasis Using a Prognostic 31-Gene Expression Profile in Subpopulations of Melanoma Patients with Favorable Outcomes by Standard Criteria. J. Am. Acad. Dermatol..

[16] Greenhaw B.N., Zitelli J.A., Brodland D.G. (2018). Estimation of Prognosis in Invasive Cutaneous Melanoma: An Independent Study of the Accuracy of a Gene Expression Profile Test. Dermatol. Surg..

