# Supplementary material for: Using a 31-Gene Expression Profile Test to Stratify Patients with Stage I–II Cutaneous Melanoma According to Recurrence Risk: Update to a Prospective, Multicenter Study
Source: Cancers (Basel). 2022 Feb 19;14(4):1060. doi: 10.3390/cancers14041060 (PMC8870692; doi:10.3390/cancers14041060)
Supplement: Supplementary file 1 [file cancers-14-01060-s001.zip › cancers-1530851-supplementary.pdf]

Supplementary Materials

Using a 31-Gene Expression Profile Test to Stratify Patients with Stage I–II Cutaneous Melanoma According to Recurrence Risk: Update to a Prospective, Multicenter Study

Sebastian Podlipnik, Aram Boada, Jose L. López-Estebaranz, Manuel M. Martín-González, Pedro Redondo, Brian Martin, Ann P. Quick, Christine N. Bailey, Sarah J. Kurley, Robert W. Cook and Susana Puig

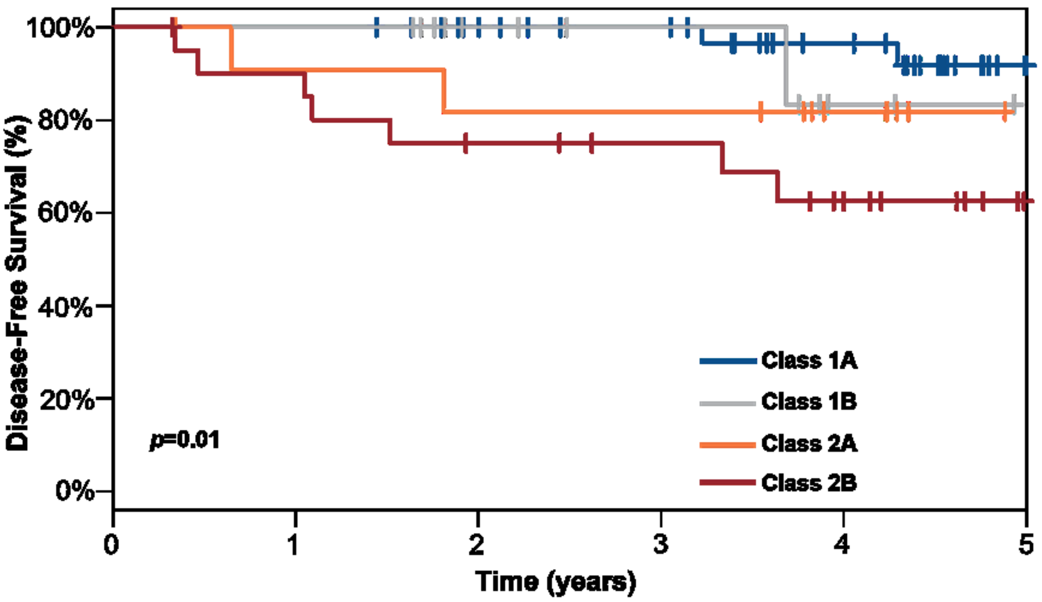

Population	3-yr DFS (95%CI)	Recurrence Rate, % (n/N)
Class 1A (*N* = 40)	100% (100-100%)	5.0% (2/40)
Class 1B (*N* = 13)	100% (100-100%)	7.7% (1/13)
Class 2A (*N* = 12)	81.8% (61.9-100%)	16.7% (2/12)
Class 2B (*N* = 21)	75.0% (58.2-96.6%)	33.3% (7/21)

Figure S1. The 31-GEP stratifies patients by 3-year disease-free survival (DFS) when analyzed by 31-GEP subclass. CI: confidence interval.

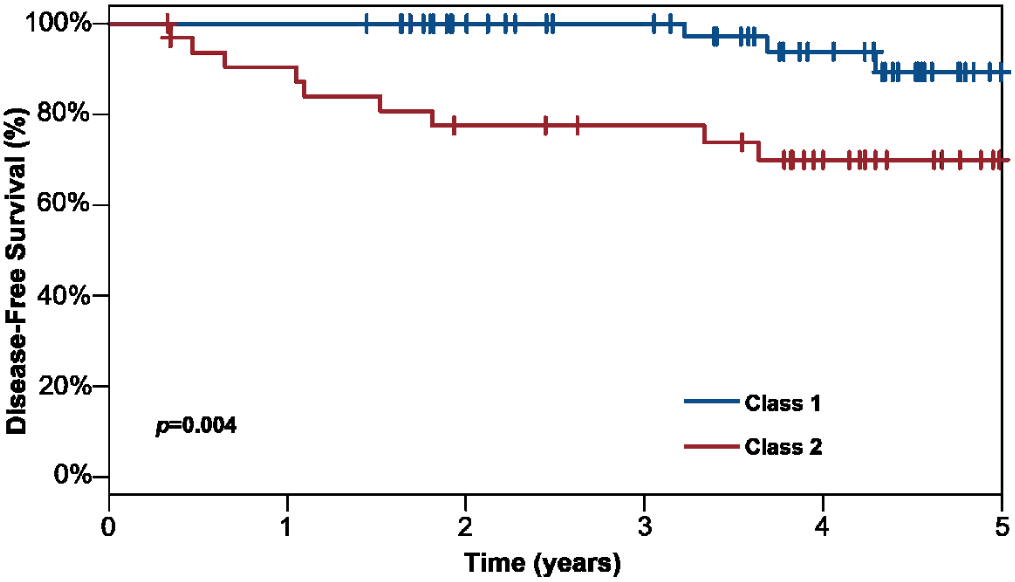

Population	3-yr DFS (95%CI)	Recurrence Rate, % (n/N)
Class 1 (*N* = 53)	100% (100-100%)	5.7% (3/53)
Class 2 (*N* = 33)	77.5% (64.1-93.7%)	27.3% (9/33)

Figure S2. The 31-GEP stratifies patients by 3-year disease-free survival (DFS) when analyzed by 31-GEP main class.

© 2022 by the authors. Licensee MDPI, Basel, Switzerland. This article is an open access article distributed under the terms and conditions of the Creative Commons Attribution (CC BY) license (http://creativecommons.org/licenses/by/4.0/).
